# Engaging patient and community stakeholders in the optimization of the Compassionate And Loving Mindset towards heart health risk (CALM Hearts) physical activity intervention: a description of initial work and protocol for future engagement activities

**DOI:** 10.1186/s40900-024-00577-z

**Published:** 2024-05-01

**Authors:** Anna Maria Chudyk, Sasha Kullman, Donna Pool, Todd Ashley Duhamel, Maureen Ashe, Shaelyn Strachan

**Affiliations:** 1 College of Pharmacy, Rady Faculty of Health Sciences, University of Manitoba, Winnipeg, MB Canada; 2 Faculty of Kinesiology and Recreation Management, University of Manitoba, Winnipeg, MB Canada; 3 Patient Partner, Winnipeg, MB Canada; 4 Institute of Cardiovascular Sciences, St. Boniface General Hospital Albrechtsen Research Centre, Winnipeg, MB Canada; 5 Department of Family Practice, The University of British Columbia (UBC), Vancouver, BC Canada

**Keywords:** Stakeholder engagement, Patient and public involvement, Patient engagement in research, Optimization, Behaviour change, Self-compassion, Participatory design, Intervention development, Protocol

## Abstract

**Background:**

Participatory research approaches systematically integrate the perspectives of individuals, organizations, or communities that have a direct interest in a study’s processes and outcomes (i.e., stakeholders) in research design and implementation. This supports interventions that are developed “by, not for” end-users, thereby increasing acceptability, uptake, and adherence. However, participatory approaches are relatively under-utilized in intervention development and behavioral change intervention research, in part, due to inadequate reporting of methodology. Therefore, to improve transparency in planning and reporting, we (a) describe how we engaged patients and community organizations (i.e., patient and community partners) in grant development for a self-compassion and physical activity behaviour change intervention for women with cardiovascular risk factors and (b) present a protocol for engaging patient and community partners in the optimization and implementation of the intervention moving forward.

**Methods:**

Our participatory research approach was guided by the Strategy for Patient-Oriented Research patient engagement framework and our prior stakeholder engagement work. Four patients and three community partners were engaged at the level of Involve, meaning their perspectives informed directions, processes, and decisions at major project milestones. Specifically, patient and community partners engaged in three separate meetings during grant development wherein they: (a) established a Terms of Reference to guide engagement activities and expectations; (b) shaped the grant through guided conversations about research priorities, outcomes, and intervention delivery components that could be targeted for optimization and (c) co-developed a protocol that specifies how relationships will be initiated with future patient partners, proposes engagement activities across the research cycle, and includes plans for formal evaluation of engagement processes.

**Conclusions:**

Participatory research approaches provide valuable insights into the development of behavioural interventions, especially when stakeholders can partner early and have a meaningful impact. By detailing our engagement activities to date, we hope to model an approach to engaging stakeholders in behavioral intervention development and demonstrate the impacts of doing so.

**Supplementary Information:**

The online version contains supplementary material available at 10.1186/s40900-024-00577-z.

## Abstract (plain language summary)

**Background:**

Participatory research engages individuals, organizations, or communities affected by a study’s outcomes (“stakeholders”), in its design and conduct. Participatory research approaches are not commonly used in intervention development and behavioral intervention research, in part due to a lack of studies describing ways to engage stakeholders in this work. Therefore, our study aimed to (a) describe how we engaged patients and community organizations (“patient and community partners”) in developing a grant application for a behaviour intervention for women with cardiovascular risk factors and (b) present a protocol for engaging patient and community partners in the future intervention.

**Methods:**

Our approach was guided by the Strategy for Patient-Oriented Research patient engagement framework and our prior engagement work. Four patient and three community partners were engaged at the level of Involve, meaning their perspectives informed directions, processes, and decisions at project milestones. Across three sets of meetings, patient and community partners: (a) established a Terms of Reference to guide engagement activities and expectations; (b) shaped the grant through guided conversations about research priorities, outcomes, and intervention design; and (c) co-developed a protocol that described how relationships will be initiated with future patient partners, potential engagement activities across the research cycle, and evaluation plans.

**Conclusions:**

Participatory research approaches provide valuable insights into the development of behavioural interventions, especially when stakeholders can partner early and have a meaningful impact. By detailing our engagement activities, we hope to model an approach to engaging stakeholders in behavioral intervention development and demonstrate the impacts of doing so.

**Supplementary Information:**

The online version contains supplementary material available at 10.1186/s40900-024-00577-z.

## Background

Behavioural science researchers often rely upon behaviour-change theory when developing and refining health-promoting interventions [1–3]. Although theory is recommended for intervention development [4], considering theory when designing an intervention does not guarantee it will be scalable from the laboratory to the “real world” or provide what end-users need. Systematic integration of the perspectives of the individuals who would participate in or implement the intervention (e.g., patients, healthcare, or community organizations) contributes to the creation of health interventions that are developed “by, not for” end users, likely improving intervention uptake [5]. Examples of partnership approaches to intervention design, which involve end-users in decision-making about the intervention throughout the development process, include co-production, co-creation, co-design, user-driven, and experience-based co-design [6]. Although these approaches hold significant promise for integrating end-user perspectives into intervention design, they are still relatively under-utilized in intervention development [6] and behavioral change intervention research [7].

The terminology and methodology of partnership approaches to research vary by country and research field, including the types of end users and other stakeholders (i.e., “individuals, organizations, or communities that have a direct interest in the process and outcomes of a project, research, or policy endeavor” [8]) that are engaged in the research partnerships [9, 10]. What these partnership approaches generally have in common, however, is a bi-directional relationship between the academic researcher and stakeholder(s), in which stakeholder(s) influence the decision-making within the research [9, 10]. This convergence of multiple perspectives sets the stage for the creation of outputs that are influenced by both scientific and pragmatic considerations [11]. Consequently, partnership approaches have been shown to contribute to improvements in research processes and outcomes, including making interventions more effective and beneficial to research partners (e.g., satisfaction with the research, widening of perspectives) and health and health systems (e.g., improved research uptake, enhanced health outcomes and decision-making) [6, 10, 12, 13]. Clearly, partnership approaches could support the development of behavioural interventions. However, the details of how stakeholders are engaged are often poorly reported [10, 14], and there is a relative lack of literature describing the application of partnership approaches to intervention development [6, 7].

Our research group is developing an intervention to increase physical activity among women at risk for cardiovascular disease (CVD). We define cardiovascular risk according to the CANHEART Index [15] as simultaneously having three or more of the following risk factors: low physical activity, hypertension, current/recent smoking, overweight/obesity, low fruit and vegetable consumption, and or diabetes. Our approach involves teaching this population to be self-compassionate (a kind and supportive way to relate to themselves), and inviting them to apply this skill to their experience of CVD risk and efforts to increase their physical activity. We call our intervention the Compassionate And Loving Mindset towards heart health risk (CALM Hearts) intervention [16]. The CALM Hearts intervention consists of three one-on-one sessions, lasting between 60 to 90 min, that are facilitated over Zoom videoconferencing. The sessions are led by a research assistant with training in self-compassion and are scheduled to occur once per week for three consecutive weeks. In the first session, participants learn about their CVD risk status, set a health behaviour goal using the SMART goals framework [17], and are introduced to the concept of self-compassion. In the second and third sessions, participants continue to receive self-compassion psychoeducation and are guided to apply self-compassion to their CVD risk and health behaviour change efforts. Between the weekly sessions, participants are tasked with making independent progress towards their health behaviour goals and completing 10–15 min of self-compassionate writing activities.

We employ aspects of the Multiphase Optimization Strategy (MOST) and the ORBIT Model to guide the development of our intervention with the ultimate aim of creating an intervention that can succeed within the community [18, 19]. Both MOST and the ORBIT Model outline an iterative process of intervention development, whereby an intervention protocol is optimized in preparation for efficacy testing and community implementation [18, 19]. For summary figures of MOST and the ORBIT Model, see the work of Wells et al. [19] and Czajkowski et al. [18]. We first considered MOST and ORBIT’s recommendations for stage 1 (preparation / define) to draw upon theoretical and empirical evidence to identify a variable upon which to intervene [20, 21]; we chose to focus our intervention on self-compassion. Self-compassion is theorized to help people cope and self-regulate their behaviour during challenging times through the provision of self-kindness and support (versus criticism), a sense that one is not alone in their challenges (as opposed to isolated) and the skill of being mindful of one’s thoughts and emotions (versus overidentification or denial) [22]. Self-compassion has been associated with health behaviours, including physical activity, and systematic reviews demonstrate self-compassion interventions can lead to changes in health behaviours [23–25]. In our own research, we found self-compassion was associated with objectively measured physical activity and adaptive emotional, cognitive, and behavioural reactions among women who learned they had a moderate to high risk of CVD [20]. However, a scoping review we conducted revealed little research (and no intervention studies) on self-compassion among those at risk for or with CVD [21].

This promising body of research led us to create and pilot test the CALM Hearts intervention among 11 women at risk for CVD. The intervention led to a 2.4-fold increase in weekly minutes of moderate-to-vigorous physical activity—a clinically significant change that provided proof of concept for the intervention [18]. Furthermore, participants found this intervention to be acceptable, but interviews with participants and community partners identified some aspects of the intervention that could be improved to increase feasibility, especially for community delivery [26]. The project’s current state of intervention development closely resembles stage two of MOST (Optimization) or phase 1b of ORBIT (Refine) where our theoretically sound and acceptable intervention will be further refined for feasibility and effectiveness. Through optimization, we will identify how to deliver the intervention effectively within the practical constraints (e.g., time, available resources) inherent to community delivery. Drawing on our previous work and the research partnership literature, we recognize that this process could be enhanced by engaging key stakeholder groups, namely patients and community organizations that could ultimately deliver the intervention, in optimizing the intervention protocol.

### Aims

Given the limited number of studies describing the application of partnership approaches to behaviour change intervention development, the first aim of this paper is to detail how we engaged patients and community organizations in developing a project grant proposal to fund the optimization of the CALM Hearts intervention. Our second aim is to present a protocol for how we will engage patients and community organizations in the optimization and implementation of the intervention moving forward. By meeting these aims, we strive to improve transparency in planning and reporting of partnership approaches to research and support the wider adoption of stakeholder engagement during the development of behavior change interventions.

## Materials and methods

This paper’s reporting is guided by the GRIPP-2 long form (Supplementary File 1).

### Conceptual framework

The Strategy for Patient-Oriented Research (SPOR) Patient Engagement Framework [27] and our previous engagement work [12, 13, 28] guided how we built and maintained relationships with stakeholders and the activities that we used to engage them, as previously conceptualized elsewhere [29]. In accordance with the SPOR Patient Engagement Framework, we use the term patient partner to refer to the patients that we engage in our research [27]. In addition, we use the term community partner when referring to community organizations that we engaged in the research process. It should be noted that although ‘stakeholder’ and ‘patient partner’ are commonly used research terms, their connotations vary among different segments of the population and at the individual level [30, 31]. Thus, it is imperative that each research team decides upon the terminology that they will use in conversation with the individuals they are engaging. We applied the spectrum of participation developed by the International Association for Public Participation [32] and adapted by Manafo et al. [33] to classify patient and community partners’ level of engagement within the study according to the amount of influence they had on the decisions made within the study. In accordance with this spectrum, we have, and will continue to, engage both groups at the level of “Involve”, meaning that we commit to working directly with both groups so as to thoroughly understand their perspectives and use this knowledge to inform study directions, processes, and decisions at all major study milestones. Since the principal investigator (SS) will have the final say in decision making, we will ensure accountability and transparency by clearly tracking all input made by patient and community partners throughout the engagement process and explaining to them how and why their input influenced study directions. Further details of engagement activities are provided below.

### Two-phased engagement approach

**Fig. 1:**
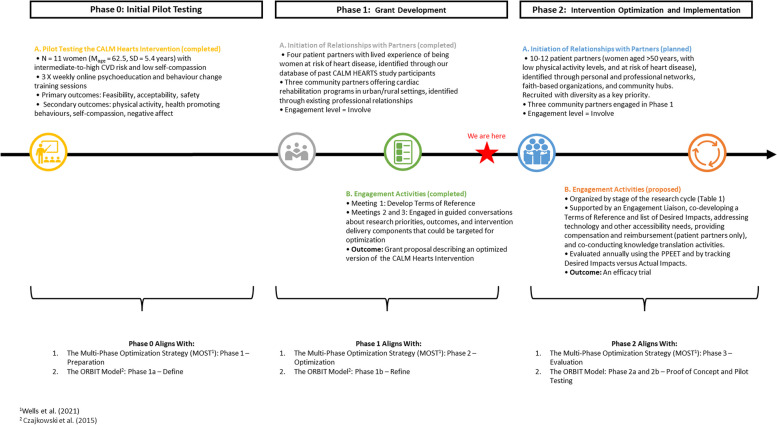
Overview of patient and community partner engagement

The grant development process represents an opportunity for stakeholders to have a meaningful impact on project directions. However, there is typically a significant time lapse between grant development and funding, during which stakeholders’ circumstances, abilities, and interests in engaging in research may change. There are also often more limited financial resources with which to engage stakeholders during grant development. This is particularly of concern when engaging patient partners as there is increasing recognition that this stakeholder group should be compensated for engagement [34]. Patient partner compensation can be monetary or non-monetary (e.g., opportunities for co-authorship, supporting desired skills training opportunities) and should be agreed upon through discussion between patient partners and academic researchers. Consequently, we engaged patient and community partners in two interconnected but distinct phases within this project (Fig. 1) – Phase 1 (completed) aimed to support the development of a grant application to fund the optimization of the CALM Hearts intervention and Phase 2 will support the processes of optimization and intervention implementation. Research ethics board approval was not obtained for either phase as we did not collect participant data but rather describe the participatory process used to develop a grant application and protocol for future engagement activities.

## Phase 1: Stakeholder engagement activities to date

### Phase 1: Initiation of relationships with patient partners

We identified the names of potential patient partners through our database of past CALM Hearts pilot feasibility study (HE2021-0175) participants who consented to being contacted about future research (*n* = 5). We contacted these individuals via an email that provided a brief overview of our aim to involve patient partners in the development of a project grant proposal. Those expressing an initial interest in involvement (*n* = 4) were sent a document containing more detailed information about the grant and study (i.e., background, methods, a description of their potential role as a patient partner, compensation, communication plans, and grant timeline. We also supported patient partners’ involvement by appointing an Engagement Liaison (SK). SK acted as a bridge between the research team and patient partners, and was responsible for ensuring the integrity of the engagement process. SK contacted each of the interested individuals via telephone or virtually (e.g., Zoom) to answer any outstanding questions. Although we had originally planned to recruit only two-to-three patient partners at this phase, we decided to enroll all four as each patient partner demonstrated enthusiasm for the project, and the inclusion of more patient voices could provide valuable insights during the grant writing process. As past participants of the CALM Hearts intervention, patient partner demographics are captured by the CALM Hearts inclusion criteria. Specifically, patient partners were individuals between the ages of 55 to 75 years, who identified as women, and had a moderate to high Framingham CVD risk score [35]. Each patient partner was provided with a $100 honorarium for their involvement in the development of the project grant.

### Phase 1: Initiation of relationships with community partners

We identified three sets of community partners through the professional networks of authors SS and TD. These community partners were deemed relevant because they represented both rural and urban communities, frequently worked with members of our target population (i.e., women at risk of CVD), and had previously established strong working relationships with members of our research team. We intentionally contacted stakeholders from both urban and rural communities to ensure our intervention end-product would be acceptable to professionals in a variety of settings. Our aim is that our community partners, or other organizations who are similar to our community partners, may one day implement the CALM Hearts program after it progresses through further stages of development. Due to scheduling conflicts, only two sets of community partners were formally engaged, though all three indicated their interest. Specifically, we engaged two administrators from an urban medically supervised exercise facility and a practitioner that provided cardiac rehabilitation services to a small, rurally located city. The research team scheduled separate meetings with the urban and rural community partners to provide more detailed information about the grant and study (i.e., background, methods, a description of their role as a community partner, communication plans, and grant timeline).

### Phase 1: Engagement of patient and community partners in grant development

Based on our previous experiences, we decided to engage patient partners together as a group (which we refer to as a patient advisory group) to give them the opportunity to form peer-to-peer bonds that support positive engagement experiences, explore and build off each other’s perspectives, and support the generation of more effective and congruent input to guide decision-making. We engaged the urban and rural community partners separately due to scheduling and availability.

Three, separate, hour-long introductory meetings were held with the patient advisory, urban community partner, and rural community partner groups. In these meetings, the groups completed ten minutes of icebreaker activities to begin fostering working relationships. Next, SK and SS introduced the history of the CALM Hearts intervention, including the names of the research team members their roles in the study, an overview of preliminary work, and the rationale for the proposed project grant. Afterwards, SK guided the patient advisory groups and community partners to develop a “Terms of Reference” document based on our previous work [29]. The Terms of Reference was a living document that guided the activities and expectations of patient and community partner engagement in the research. SK facilitated the development of this document by posing questions to the patient advisory and community partners and recording their answers. Specifically, each partner stated their needs for a safe work environment; defined what a meaningful contribution to the project would mean, look, and feel like; identified practical supports that should be provided by the research team (i.e., information, technology, education, etc.); and stated their preferred method and frequency of communication with the research team (Supplementary Tables 2– 4).

In developing the Terms of Reference, the patient advisory group identified two supports that would facilitate their involvement in the project grant. First, they required more information about the research team’s expectations of patient partners. Second, they requested a timeline of grant development that identified specific opportunities for patient partner involvement. The urban community partner requested that the research team provide detailed progress summaries at the start of each team meeting to serve as a reminder of the project’s goals and directions. They also requested that the eventual research findings be shared with their facility’s members and staff through infographics, newsletters, or presentations. The rural community partner requested that the research team provide more information about any specialized training required to administer the CALM Hearts intervention. The rural community partner also indicated an interest in being informed about aspects of methodological decision-making such as study design, development of documents, and participant eligibility criteria. Each group’s completed Terms of Reference document was sent to them within one week of their introductory meeting.

Next, the patient advisory group and community partners were involved in developing the grant proposal. To this end, SS and SK first engaged the patient advisory group in two, hour-long meetings, which shaped our plans to optimize the CALM Hearts intervention and ensured the grant reflected patient-identified priorities and outcomes. Ahead of the meetings, patient advisory group members received a document listing 13 intervention delivery components that could be targeted for optimization. In the two meetings, the patient advisory group considered the following questions: *(1) Which of the 13 intervention delivery components do you feel are most important to change? (2) Which changes would have most improved your experience as a research participant? (3) Are there any aspects of the intervention not listed that we should improve?* The advisory group’s responses to these questions informed priority areas for optimization which we subsequently presented to our community partners. We engaged community partners in a similar process of identifying optimization targets as described above.

Based on the patient advisory group’s and community partners’ feedback, and in discussion with the remainder of the research team, we pursued the following optimization targets. First, we aim to change the intervention delivery model from individual to group based. The patient advisory group noted that hearing how others benefit from and use self-compassion may aid in self-compassion adoption. Community partners confirmed that their current programming was often delivered in a group format, and optimizing the CALM Hearts program for group delivery would promote an easier transition to community implementation. In further support of group delivery, systematic reviews and clinical research show both individual and group delivery methods have similar efficacy in health behaviour interventions [36, 37]. Group delivery may also be well suited to self-compassion interventions since supportive social interactions can foster self-compassion [38, 39] and meta-analytic results show the superiority of group-based self-compassion interventions at increasing self-compassion [40]. Given patient and community partners could see value in group delivery and given support for group delivery of behaviour change programs, including self-compassion programs, we will deliver the intervention to small groups in a subsequent trial.

Second, the patient advisory group suggested the inclusion of a “booster” intervention session. This session would be delivered at a time interval (e.g., one month) after the conclusion of the core intervention. In explaining their desire for a booster session, patient partners stated that the interval between the conclusion of the main intervention sessions and the booster session would allow participants to practice their physical activity self-management skills independently, knowing they could return for group support at the time of the booster session. The booster would be an opportunity for participants to seek support in addressing barriers to their physical activity change. Community partners saw value in the addition of a booster session but suggested the research team should seek evidence for the benefit of a booster before their organizations committed additional resources to this extra session. Findings about the impact of boosters on physical activity are mixed [41, 42], but a self-compassion intervention including a booster achieved change in outcomes including physical activity [43]. The use of booster sessions in self-compassion interventions has been suggested by participants [44] and researchers [45]; therefore in a future trial, we will determine the ideal timing for, and test the benefits of a booster session within our optimized intervention.

Finally, discussion with community partners led to a unique addition to the intervention. The research team discussed with them the training of centre staff to deliver the intervention; to date it had been delivered by research assistants. Further, the research team believed that to optimize the intervention for community delivery, centre staff should be trained to deliver it. Discussions with both sets of community partners led to the development of a plan to involve centre staff in creation of and feedback on a training plan and ultimately, a training manual.

The resultant impact of patient and community partner engagement on the project grant application and proposed study directions are summarized in Supplementary File 2. Both the patient and community partners agreed to future engagement requests as the project progressed. However, patient partners stated that their agreement to engage would depend on their availability. One patient partner (DP) indicated interest in co-authoring this paper. The same patient partner and all of the community partners indicated interest in co-developing the Phase 2 engagement protocol presented next.

## Protocol for phase 2 engagement activities

### Phase 2: Initiating relationships with patient partners and community stakeholders

When we acquire grant funding for this project, we will continue working with our existing community partners and re-initiate and develop new relationships with patient partners in the 4–6 months prior to the start of the CALM Hearts intervention. We will aim to assemble a patient advisory group of 10–12 patient partners to guide the study across the remainder of its research cycle. This number is based upon our previous experiences with patient advisory groups [29, 46], group dynamics [47], and to account for the potential loss of patient partners over time. To help maximize the odds of patient partners being able to attend advisory group meetings, we will aim to send out meeting scheduling polls at least two weeks in advance of targeted meeting dates. Generally speaking, our source population of patient partners (women at risk of CVD) is relatively healthy, but may have competing interests (e.g., work or family responsibilities) that may impact on their ability to attend a given meeting. Thus, to help ensure the project meets its timelines, we plan to go forward with any given meeting as long as we meet quorum (defined as half the number of advisory group members + 1). We will make every reasonable effort to gather the perspectives of those not in attendance through scheduling alternative individual or small group meetings and offering the opportunity to provide written feedback on the meeting topics.

We will identify patient partners using multiple strategies, including ones targeted at women typically underrepresented in cardiac research (e.g., women who self-identify as belonging to ethnically diverse groups and of lower socioeconomic status [48]). We will also target women living in rural communities to ensure our intervention can be implemented in both rural and urban settings. Specifically, we will work with key stakeholders to help raise awareness about the study [49, 50], including personal and professional networks (e.g., patient groups, the Manitoba Primary Care Research Network, our health system – including healthcare providers, health and social service care hubs such as ACCESS Centres), and the community partners involved in grant development. We will also advertise the study within faith-based organizations and community hubs (e.g., libraries, community centres) in neighborhoods of lower socioeconomic status and with higher proportions of ethnic diverse populations if necessary to help ensure a diverse patient advisory group. Modes of advertisement may include email, websites, social media (e.g., Facebook, Twitter), posters, videos, and information sessions.

Our advertising will be targeted toward women over 50 years of age who identify as having low physical activity and high blood pressure or other cardiovascular risk factors (e.g., current/recent smoking, overweight/obesity, low fruit and vegetable consumption, and or diabetes [15]). This strategy should increase the likelihood that advisory group members will share lived experiences with intervention participants which will affect their opinions about the intervention. Individuals who participated in the initial CALM Hearts intervention will be eligible for advisory group membership, but this experience is not a prerequisite. As guided by the literature [51] and our previous experience, we will screen all individuals that express interest in advisory group membership to help ensure diversity and readiness to meaningfully engage with our research.

### Phase 2: Proposed engagement activities by stages of the research cycle

Based on our previous work [7], proposed engagement activities are organized by the stages of the research cycle (Table 1). This initial set of proposed activities was mutually developed with patient and community partners from Phase 1 grant development. Specifically, SK engaged with one patient advisory group member (DP), the urban community partner, and the rural community partner across three separate meetings. The engagement activities proposed for the patient advisory group and community partners differ slightly based on the different interests and expertise of patients and community stakeholders. However, the activities will be reviewed and potentially revised at key study points (e.g., when the Phase 2 patient advisory group is formed, as the study evolves over the stages of the research cycle) to ensure they reflect patient advisory group members’ and community partners’ interests and study needs.

**Table 1:** Proposed engagement activities across stages of the research cycle

Stage	Proposed activities
Revising the grant	♦ Provide input on priority areas for intervention optimization
Designing the intervention	♦ Help to develop or revise aspects of the intervention by identifying practical concerns ♦ Participate in focus groups ♦ Engage in meetings with the core research teams and/or other stakeholder groups engaged in the project
Choosing outcomes and how to measure them	• Provide insights on issues and outcomes that matter to patients • Work with academic researchers to collaboratively select specific outcome measures (e.g., psychological scales), review questionnaires for comprehension and clarity, advise academic researchers on whether certain outcomes should be measured qualitatively, quantitatively, or both ▪ Provide insights on issues and outcomes that matter to community stakeholders ▪ Review and provide feedback on the measures selected to ensure these measures meet community partners’ information needs • Share experiences with the study topic
Helping to develop or revise study materials	• Help to develop or revise the study materials (e.g., visual appeal, wording, timing) • Prepare other patients to provide input on the study materials (e.g., getting feedback from similar others) ▪ Discuss whether study materials can be feasibly delivered in the community ♦ Review the plans to conduct the study (e.g., approving the timeline)
Recruitment	♦ Provide suggestions for increasing participant diversity ♦ Suggest additional locations to recruit more participants ♦ Help to discuss and determine recruitment strategies • Help to develop recruitment scripts • Help to develop patient-facing content (e.g., consent forms) ♦ Help to recruit participants through personal networks (e.g., directly contacting people, providing contact information to the research team, promoting research among networks) ♦ Present at recruitment sites on behalf of the research team
Data Collection	• Help collect qualitative data (e.g., conducting interviews with participants in the study) • Help administer online questionnaires and compile data • Pilot being a participant in the study • Participate in the study
Data Analysis	• Be part of the team that analyzes qualitative data (e.g., read interview transcripts and help create themes/meanings from interview data) • Be part of the team that analyzes quantitative data (e.g., help decide how data are summarized/displayed and what analyses are performed)
Data Interpretation	• Help to review the results and provide reflections about the data • Ensure the way the results are described is accessible to patients ▪ Ensure the way the results are described are meaningful to community stakeholders ♦ Help identify key findings and plan next steps • Participate in focus groups aimed at validating findings
Knowledge translation	♦ Provide perspectives and ideas about how we can share knowledge from our study with other groups ♦ Help to draft, revise, and co-author research papers ♦ Help to revise or draft non-manuscript written materials (e.g., newsletters, briefing notes, participant summary reports) ♦ Help to develop or revise conference submissions and presentations ♦ Share research findings through social media or other social networks • Present at conferences or attend conferences on behalf of the study team • Present in non-conference settings (e.g., interviews or townhalls, podcasts, videos)

•Patient advisory group activity; ▪Community partner activity; ♦Patient and community partner activity

We will support patient advisory group members and community partners in engaging to the best of their capacity through (a) appointing a liaison responsible for ensuring the integrity of the engagement process, (b) co-developing a Terms of Reference that will guide patient and community partner conduct, (c) creating a list of desired impacts of engagement that are valued by members of the patient advisory group, community partners, and the broader team, (d) administering a self-developed survey aimed at identifying technology and other accessibility needs, (e) providing compensation and re-imbursement the for the patient group’s time, expertise, and expenses incurred during engagement and (f) and conducting knowledge translation activities with patient and community partners to ensure our research has a positive impact on their communities and organizations.

### Phase 2: Evaluation of engagement

We will evaluate both the quality and impact of engagement in our study. Specifically, we will evaluate the quality of our engagement activities on a yearly basis, from the perspectives of members of the patient advisory group, community partners, and broader research team, using the Public and Patient Engagement Evaluation Tool (PPEET) for long-term engagement initiatives [52]. This generic, three-part tool is often used in health research to evaluate key elements of quality engagement including the integrity of the design and process, influence and impact, participatory culture, and collaboration and common purpose [52]. A recent review found that the PPEET was developed using a scientifically rigorous process, took into account the views of patients and the public, comprehensively evaluated engagement, and was easy to use [53]. In addition, to help ensure that engagement achieves the impacts that are valued by both members of the patient advisory group and the broader team, we will co-develop a working list of desired impacts. Desired impacts will be organized by time frame (i.e., immediate/near-term, intermediate, and long-term impacts) [12], level of influence (i.e., value to patients, value to community stakeholders, value to researchers, improvements to research processes, impact on policies and decisions, impact on health outcomes, contributions to social change in research) [54], and stage of the research cycle. The achievement of these impacts will be evaluated at key study points through discussion with the patient advisory group and community partners.

## Discussion

Partnership approaches to research can provide valuable insights into the design and evaluation of behavioural interventions, especially when stakeholders are provided with the opportunity to partner early and have a meaningful impact. However, partnership approaches to intervention design are under-utilized in intervention development [6] and behavioral intervention research [7]. Therefore, this work has the potential to make both immediate and more downstream contributions to the field. By detailing our engagement activities to date, we hope to model an approach to engaging patient and community stakeholders in behavioral intervention development and demonstrate the impacts of doing so. The considerations we identify throughout this work should act as a resource to support the more widespread engagement of stakeholders in health research. Finally, the publication of protocols such as this, addresses the lack of transparency in the planning and reporting of stakeholder engagement observed in the current literature [55].

When considering our engagement approach, some limitations warrant mention. First, where stakeholders are engaged on the spectrum of participation [32, 33] influences the impact they will have on the decisions made within the study. As our engagement occurs at the level of Involve, we have committed to working directly with stakeholders throughout the study’s research cycle, so that their perspective will help inform the decisions made about the study. Both of our patient and community partner groups will be engaged throughout the study’s research cycle to ensure that their perspectives are understood and considered as decisions are made throughout the course of the study. However, the study’s ultimate decision-making power lies with the principal investigator (SS), which could potentially lead to tokenistic engagement if proper mechanisms are not in place to prevent this. Thus, we have committed to transparency and accountability within the engagement process through regular documentation of stakeholder inputs and how they influenced project directions (and if not then why). The Engagement Liaison (SK) also helped to ensure the integrity of the engagement process and upheld partner interests during Phase 1 of engagement. Further contributing to accountability are the Terms of Reference and engagement planning documents that will record the ways in which patient and community partners would like to engage, the types of impacts they hope to have, and an annual evaluation of engagement activities. Another limitation is that our engagement activities to date included only four patient partners, which may have resulted in input that does not reflect the experiences of voices less typically heard in women’s cardiovascular research (e.g., women from ethnically diverse groups or of low socioeconomic status). The patient and community partners that we engage in the future will have the opportunity to actively shape the evolving research project, including their roles and responsibilities within it. Relatedly, we did not obtain ethics approval to report on the demographics of patient partners in this manuscript, as our aim was to describe the participatory process used to develop a grant application and protocol for future engagement activities. Doing so would have allowed us to help promote transparency and diversity by better demonstrating whose voices are represented and whose are not in our research.

## Conclusions

This protocol outlines approaches to patient and community stakeholder engagement in both the development of a project grant, and in the future optimization and implementation of the CALM Hearts intervention. This work is timely given that partnership approaches are under-utilized in behavioural interventions [7]. We note important considerations that may help guide other behavioral intervention researchers in incorporating stakeholder engagement within their work. Ultimately, we aim to develop an intervention that can help women relate to themselves with more compassion as they increase their physical activity and reduce their CVD risk. By embedding stakeholder engagement in our intervention development and optimization process, the perspectives of the population that will benefit from the intervention and community groups that will ultimately deliver it will play a key role in bringing these goals to fruition.

### Supplementary Information

**Additional file 1: Supplementary File 1.** GRIPP2 long form.

**Additional file 2: Supplementary Table 1.** Patient and community partner suggestions and resulting impact on project grant proposal.

**Additional file 3: Supplemental Table 2.** Patient Partner Terms of Reference. **Supplemental Table 3.** Urban Community Partner Terms of Reference. **Supplemental Table 4.** Rural Community Partner Terms of Reference.

## Data Availability

No datasets were generated or analysed during the current study.

## Abbreviations

CALM Hearts: Compassionate And Loving Mindset towards heart health risk
CVD: Cardiovascular disease
MOST: Multiphase Optimization Strategy
ORBIT: Obesity Related Behavioral Intervention Trials (ORBIT) Consortium
PPEET: Public and Patient Engagement Evaluation Tool
SPOR: Strategy for Patient-Oriented Research
