# Supplementary material for: Engaging patient and community stakeholders in the optimization of the Compassionate And Loving Mindset towards heart health risk (CALM Hearts) physical activity intervention: a description of initial work and protocol for future engagement activities
Source: Res Involv Engagem. 2024 May 1;10:42. doi: 10.1186/s40900-024-00577-z (PMC11062915; doi:10.1186/s40900-024-00577-z)
Supplement: Supplementary file 2 — Additional file 2: Supplementary Table 1. Patient and community partner suggestions and resulting impact on project grant proposal. [file 40900_2024_577_MOESM2_ESM.docx]

Supplementary Table 1. Patient and community partner suggestions and resulting impact on project grant proposal.

| **Framing Question** | **Feedback from Patient and Community Partners** | **Result** |
| --- | --- | --- |
| **Recruitment**  As research participants, what are the main barriers to entry and/or participation in our intervention that you can identify? | **Need to increase awareness among patients**   - Advertise the study in doctor’s offices. - Work with regional health agencies to recruit and spread awareness.   **Need increased representation from diverse groups**   - Current group of research participants is too homogenous (i.e., white, middle class). - Increase the ethnic diversity of participants to represent the diversity within the province. - Recruit from both urban and rural areas to ensure a far-reaching intervention. - Do certain recruitment methods lead to wider representation? – experiment and compare methods.   **Need to address technological barriers**   - Barriers may include personal comfort, little experience with the internet, or lack of access. - The technology requirements of online delivery can skew the education/cultural background of participants.   **Need to acknowledge cultural barriers to accepting self-compassion**   - Low comfort with directing focus/compassion towards self. - Fear of self-compassion. - Possibly approaching cultural groups to find ways to tailor program to beliefs and values (culturally relevant programming). - Make intervention product more generic so it can be tailored to diverse people and cultures. - Language adjustments – how can self-compassion be discussed in a meaningful way for diverse groups? | **Increasing awareness**   - We will share all recruitment material with the patient advisory group for review and feedback. - We will work with the regional health agency to recruit participants and spread awareness.   **Increasing representation from diverse groups**   - We will seek out community leaders from organizations representing diverse groups to spread awareness about the study. - We will increase the diversity represented on our future patient advisory panel for the full trial.   **Addressing technological barriers**   - We will offer training prior to the start of the intervention to orient participants to the online platforms. - We will ensure the intervention materials are flexible such that the intervention can be delivered either virtually or in person.   **Addressing cultural barriers to accepting self-compassion**   - We will seek the guidance of ethnically/culturally diverse members of our future patient advisory panel to ensure our participant-facing materials are appropriate. - Fears and misgivings about self-compassion will be addressed in the first session and participants will have an opportunity to discuss. - The intervention facilitator will invite participants to call self-compassion by a different name if doing so makes them feel more comfortable about this topic. |
| **Discussions**  In the intervention sessions, we currently frame discussions and  activities around applying self-compassion to your risk for heart disease and the challenges  of becoming more physically active. Did you find it useful to apply self-compassion  in this way, or would you have preferred a more generic discussion of self-compassion? | **Applying self-compassion to specific topics (i.e., heart disease risk and physical activity) was helpful for participants’ learning.**   - Self-compassion can feel like an overwhelming topic at first, but having a specific focus can make it feel more accessible. - Applying self-compassion to physical activity and heart disease risk allows time to develop self-compassion skills. These skills can then be applied to other areas of life. - Framing self-compassion in the context of heart health can reduce some of the taboo around treating oneself with kindness. - It is important to remember that the purpose of the intervention is to apply self-compassion to the specific situations of heart disease risk and physical activity. The discussions in the session should reflect this. | **Framing discussions in the future intervention**   - The intervention will continue to frame discussions of self-compassion around heart disease risk and physical activity. - Some changes may be made in the future based on input from patient advisory group members representing more diverse groups. |
| **Delivery Mode**  Should the intervention sessions be delivered one-on-one to each  participant, or to small groups of 6-9 participants.  Prompts:   - Were there benefits of receiving the intervention   individually that would be lost in a group setting?   - Would there be anything more to gain from experiencing   the intervention in a group? | **Benefits of one-on-one delivery**   - It can be easier to express one’s feelings in a private setting. - More time and individual attention can increase the depth of discussion between the participant and facilitator. - One-on-one attention may be better when setting physical activity goals and asking for individualized feedback from the facilitator.   **Potential benefits of group sessions**   - Other group members can share examples of how they apply self-compassion to their heart health and physical activity. - Other participants can serve as a good reminder that many people struggle with their heart health and physical activity. - Listening and learning from others can foster common humanity (an aspect of self-compassion that reminds people they are not alone in their struggles). - Group delivery is more time and resource efficient. - Group delivery may be more acceptable for delivering the intervention in the community.   **Suggestions for a hybrid one-on-one / group delivery format**   - The first and last session could be delivered individually to allow participants to discuss their goal progress. - The middle sessions could be delivered in a group to facilitate group-learning. - The intervention could be delivered as an individual workbook-based program with individual or group meetings interspersed throughout the weeks. | **Decision regarding delivery method**   - Despite some drawbacks of losing the private nature of one-on-one delivery, group delivery seems appealing to participants. - Group delivery should also be more acceptable to community organizations who may not have the time or resources to deliver programming individually to each client. Therefore, we will switch the intervention to a group delivery model. |
| **Physical Activity**  Should we provide self-compassion education alone, or also include opportunities to be physically active as part of the intervention sessions? | **The current classroom style intervention (without physical activity component) was helpful:**   - Ensures participants think of their own systems for staying active outside of the sessions. - Participants have time to work on their physical activity independently between sessions and then report back on their progress. - Exercise between classroom sessions can be incorporated into every-day life, and does not need to be structured into a time of day/location. - Providing physical activity behavior change information in addition to self-compassion education might be helpful to support participants in their behavior change.   **Possible suggestions for incorporating physical activity into sessions**   - Participants could be encouraged to walk in place while attending intervention sessions. - The facilitator could provide online links to exercise resources. - Facilitators should ensure exercise resources are appropriate to physical limitations. - Walking with the intervention facilitator during in-person sessions. - Providing participants with a gym membership.   **Drawbacks to incorporating physical activity into the sessions**   - Weather-related barriers to exercise (e.g., walking outside). - Transportation barriers. - Participants may not think of their own systems for staying active outside of the sessions. - Too much structure – participants may not find the time of day the sessions are delivered ideal for exercise.   **Takeaway message**   - Anything that could be helpful to get people moving is worthwhile. | **Decision regarding inclusion of physical activity component**   - There seem to be advantages to maintaining a classroom style intervention and encouraging participants to engage in physical activity outside of the sessions. Therefore, we will keep our current classroom-style delivery method. - We will provide more physical activity resources to participants in the form of a website that lists links to appropriate guided physical activity videos, reading materials, podcasts, and other health information. - We will spend more time in the first intervention session discussion physical activity, the physical activity guidelines, and strategies for incorporating physical activity into one’s life. - Participants will be encouraged throughout the sessions to evaluate the progress made towards their physical activity goals. - While we will not develop additional content relating to behaviour change strategies, we will keep the format of our intervention flexible such that it can be incorporated into existing health behaviour change interventions delivered by community organizations. This should allow our self-compassion content to be easily absorbed into existing programming. |
| **Session Timing and Number**  We currently offer four, 60-minute intervention sessions.  Were you satisfied with the number and length of sessions provided in the intervention?  Prompts:   - Did you feel that there was sufficient time to engage in the discussions and activities? - Should the number of sessions be increased or decreased? | **Number of sessions could be increased, but the length of sessions was sufficient.**   - Increasing the number of sessions would provide more time for discussion and activities. - The quality of the intervention may increase if the number of sessions increased.   **A follow-up (“booster”) session would be beneficial for continuity and support after the intervention concludes.**   - Participants felt a sense of “let-down” after the sessions were over because there was no follow-up to look forward to. - A follow-up meeting after the conclusion of the intervention (e.g., 1-month post-intervention) would be helpful for motivation, encouragement, and feedback on goal progress. - Research group could determine the optimal time for a follow-up session that allows participants time to practice physical activity maintenance independently, but supports habit formation by providing an opportunity to share progress and address barriers.   **Maintaining continuity of the intervention without a booster session**   - Research group could create a mailing list with resources or updates for past participants to support their physical activity. - Research group could circulate longer-term follow-up questionnaires to ask past participants about their physical activity progress.   **Participant-led initiatives for staying connected**   - Development of an online group for past research participants to join (e.g., a Facebook group). - This group would provide an opportunity to connect and check in with people who have completed the intervention. - Good source of accountability. | **One additional 60-minute session will be provided.**   - We will add one more session (4 sessions total) to provide more time to cover all content. - Future patient advisory panel will be involved in developing the discussion prompts and in-class activities that will be distributed throughout the sessions.   **Addition of a booster session**   - We will provide one follow-up session where participants can check-in with the intervention facilitator and their intervention group. - This will be an un-structured session centred around physical activity successes and barriers. - The research team must determine the optimal timing of this session and the necessity of a booster. A small pilot study with longitudinal follow-up timepoints spanning a few weeks to a few months post intervention may help to determine when participants’ physical activity levels begin to decline and participation in a booster session would be optimal. - Long-term follow-up will also benefit the research team by providing more information about the sustainability of the interventions’ effects and the necessity of a booster session.   **Participant-led initiatives for staying connected**   - The research team will discuss this with the institutional research ethics board before implementation. The intervention facilitator may be able to help interested participants stay in contact with one another but may not be permitted to engage with past research participants via social media. |
| **Session Frequency**  We currently offer the intervention sessions once per week for three weeks.  Were you satisfied with the timing of the sessions? | - Once per week was satisfactory. - Meeting on a regular day and time was helpful for planning physical activity and home-practice activities relating to the intervention. | **The timing of the sessions (once per week) will be maintained.**   - No change required. |
| **Virtual Delivery**  We currently offer the sessions virtually over zoom. Were you satisfied with participating via zoom or would you prefer in-person sessions over virtual sessions? | **Benefits of Zoom**   - Virtual meetings accommodate people who live in rural areas. - There is no need to drive to a meeting in the winter. - Opportunity for participants to learn new technology (zoom).   **Possibility of hybrid meetings**   - The research team could facilitate hybrid intervention sessions where some participants participate in-person and others participate via videoconferencing. - Hybrid sessions could encourage more individuals to participate in the study. - Participants could be offered the opportunity to meet up for a walk if they are interested in meeting in-person between videoconferencing sessions. | **Conclusions Regarding Intervention Delivery**   - Because the intervention facilitators currently reside in an urban area, but participants may come from both rural and urban areas, we will continue to host the sessions through Zoom videoconferencing. - The design of the intervention will be kept flexible such that it could be delivered either virtually or in person. This will allow future providers in the community to decide upon a delivery model (virtual or in-person) based on the needs of the population they serve. |
| **Facilitation**  A trained facilitator currently hosts the intervention sessions. Based on your experience, was the presence of a facilitator  meaningful and necessary? Do you think this intervention could be delivered as a series  of self-guided online modules or workbooks? | **Benefits of a trained facilitator**   - The facilitator is a source of meaningful human interaction throughout the intervention. - The facilitator provides accountability, guidance, and advice (when requested). - If an “independent work” component was added to the intervention, participants would still benefit from having semi-frequent meetings with a facilitator for accountability and support.   **Training center staff to deliver intervention.**   - The intervention will eventually be implemented within community stakeholder organizations. Therefore, the research team should develop materials to train center staff who will deliver the intervention in the community. | **We will continue to deliver the intervention using a trained facilitator.**   - No change required.   **Training center staff to deliver the intervention.**   - We will involve centre staff in creation of and feedback on a training plan and ultimately, a training manual for delivering the intervention. |
| **In-Class Activities**  During the intervention sessions, we ask participants to complete some writing activities in their workbooks. Based on your experience, were the in-class workbook activities helpful?  Prompts:   - Was it necessary to write in the workbook, or could the same activities be completed through discussion with the facilitator alone? - Was the online workbook acceptable, or would you prefer that a physical copy of the workbook be delivered to you? | **Writing in the workbook is helpful**   - Writing gives participants time to reflect on and interpret the material. - This is particularly important for personal topics such as self-compassion.   **Time provided for writing in sessions**   - Provide enough time to ensure participants do not feel rushed while writing in their workbooks.   **Printing the workbook**   - Printing the workbook at home or using a digital workbook may not be feasible for all participants. - Researchers should provide a physical copy of the workbook. | **Writing activities will be maintained in the intervention**   - No change required.   **Additional time will be provided for writing**   - As stated above, we will add one more 60-minute session (4 sessions total) to provide more time to cover all content. - If this is still insufficient, we will consider extending the length of each session to 90 minutes.   **Printing the workbook**   - Researchers will print the workbooks and provide hard-copies to all participants. |
| **Home-Practice Activities**  Each week between intervention sessions, participants  engage with self-compassionate writing activities in their workbooks. Based on your experience, were the home practice activities helpful?  Prompts:   - Could the home practice activities be improved? - Were the home practice activities meaningful and necessary? - Could the home practice activities be removed altogether? | **Number of home practice activities**   - The intervention assigns the right amount of home practice, in that the tasks are not overwhelming but also do not leave participants wanting more.   **Framing of activities**   - Some questions seemed leading because they were framed around applying self-compassion to feelings of isolation. When participants do not feel isolated, it can be difficult to relate. - Some participants may need more practice with self-compassion than others (a benefit of the group sessions is that participants would gain insight into the self-compassionate practices of others). - In future, the language used in the workbooks and the activities should be co-developed with patient partners to ensure they are relevant and appropriate. | **Number of home practice activities will remain the same**   - No change required.   **Activities will be revised to increase relevance to participants**   - In collaboration with patient partners, the workbook activities will be re-worded and revised. |
| **Technology**  Would a smartphone/iPad app that supports your participation in  the study be helpful? For instance, researchers could provide participants with an app that reminds them to complete home practice activities and  engage in physical activity. | **Pros and cons of incorporating technology**   - Helpful for those with the technology to support a smartphone app. - New technology can be uncomfortable or unfamiliar for some participants. - Researchers should be mindful as too many reminders can be annoying for participants. | **Alternative, low-technology, reminder system**   - A smartphone/iPad app was not seen as acceptable and will not be used in a future intervention. - Research team will remind participants to complete home practice activities using optional text-message reminders. - Participants will have a choice about receiving text message reminders and can opt out at any time. |
| **Use of PowerPoint**  The intervention is currently guided by a series of PowerPoint  presentations. Based on your experience, did you enjoy following along  with the PowerPoint slides in each intervention session?  Prompts:   - Would your experience have been better/worse/the same if each session was a flexible discussion with the facilitator instead of being guided by a PowerPoint? | **Pros of PowerPoint**   - Useful for visual learners. - Can ensure the intervention is delivered in a consistent way.   **Cons of PowerPoint**   - May be more geared towards the facilitator than the participant. - May be the only focal point of the conversation, or make the conversation too rigid.   **Alternatives**   - Reduce the number of slides, such that the PowerPoint is used as a supplement, but is not the main focal point of the sessions. - Intervention is delivered primarily through the workbook, and slides are used minimally for guidance. | **PowerPoint slides will be revised**   - We will reduce the number of slides, such that the PowerPoint is used as a supplement, but is not the main focal point of the sessions. - Future PowerPoint presentation used to guide the intervention will be developed in collaboration with patient partners to improve acceptability. |
| **Assessing Physical Activity**  We currently assess physical activity using a questionnaire. In the  future, we would like to assess physical activity using an activity  monitor called an accelerometer.  As a research participant, would you find it acceptable to wear a small, hip-worn, activity monitor during waking hours for 8 days  before and after the intervention? | **Accelerometers are acceptable**   - Wearing the accelerometer after the intervention could be a good motivator to increase physical activity. - Facilitator could incorporate accelerometer results with the first and last meeting. In this meeting, the facilitator could share individual physical activity data with participants. - Providing participants with a pedometer could also be useful for tracking physical activity. | **Use of accelerometers**   - In a future intervention we will assess physical activity using accelerometers. - Further discussion is needed about sharing individual physical activity data with participants during the intervention. Sharing this information creates concerns around participant confidentiality (if intervention is delivered in groups), and could also disrupt the fidelity of the intervention (if participants find their physical activity data motivating). |
| **Other**  Are there any other aspects of the intervention that you would like to discuss today? | **Should the research team use a clinical outcome like blood pressure to measure the efficacy of the intervention?**   - Blood pressure is relevant to clinicians. - Participants could be asked to measure their own blood pressure as a motivator for behaviour change. - Access to blood pressure cuff could be a concern. Research team could provide cuffs or ask participants to visit a pharmacy for a blood pressure reading. - Videos and education should be provided on how to take blood pressure measurement. - Monitoring blood pressure is a health behaviour, but participants may feel overwhelmed if they are asked to change too many health behaviours at once (e.g., increasing physical activity and monitoring blood pressure). | **Opportunity for further discussion**   - Monitoring blood pressure may be motivating but could present challenges due to variable accuracy of different measurement methods (e.g., home measurement, versus clinic measurement, versus pharmacy measurement). - With more time available for planning, the research team will discuss the potential for blood pressure as an eligibility criterion for the intervention. - Blood pressure could be shared with participants as a motivator for physical activity change. |
