# Supplementary material for: Engaging patient and community stakeholders in the optimization of the Compassionate And Loving Mindset towards heart health risk (CALM Hearts) physical activity intervention: a description of initial work and protocol for future engagement activities
Source: Res Involv Engagem. 2024 May 1;10:42. doi: 10.1186/s40900-024-00577-z (PMC11062915; doi:10.1186/s40900-024-00577-z)
Supplement: Supplementary file 3 — Additional file 3: Supplemental Table 2. Patient Partner Terms of Reference. Supplemental Table 3. Urban Community Partner Terms of Reference. Supplemental Table 4. Rural Community Partner Terms of Reference. [file 40900_2024_577_MOESM3_ESM.docx]

| **Supplemental Table 2**  Patient Partner Terms of Reference | | |
| --- | --- | --- |
| Item # | Question | Patient Partner Response |
| 1 | What does a safe work environment mean to you? What are your goals for this partnership? | - To work together with respect and kindness. - To foster a tolerant environment where we are open to new ideas and are safe to disagree with each other. - To create an environment where we are free to express our feelings. |
| 2 | What does a “meaningful contribution” mean / look like / feel like to you | - Engagement throughout the entire project. - Researchers should share information about the end outcomes of the study. - Patient partners should feel fulfilled from their engagement in the research project. - We may not know if we made a meaningful contribution until the end of the project when we look back at what we accomplished. |
| 3 | How can we support you in this collaboration? | - Researchers should provide more information about how specifically patient partners can help with grant development. - Researchers should create a flow chart explaining how patient partners can assist at each step of grant development. - Researchers should define what kind of feedback they are seeking from patient partners (e.g., opinions, guidance) - Researchers should provide a timeline of key meetings and milestones in the grant development process. |
| 4 | What are your preferred methods of providing feedback to the research team? | - Paper copies or email. - Verbal conversations over Zoom call. |
| 5 | How often would you like to meet? | - Meet at key milestones and/or when feedback is required. |
| 6 | How would you like to receive progress updates? | - Email or paper copies are both acceptable. |
| 7 | Is there anything else you would like to add? | NA |

| **Supplemental Table 3**  Urban Community Partner Terms of Reference | | |
| --- | --- | --- |
| Item # | Question | Community Partner Response |
| 1 | What are your goals for this partnership? | - Learn new tools for effective program delivery. - Keep current with research literature. - Spread information from this research to our fitness and health team. - Opportunity for staff to meet for informal discussion to translate research into practice. - Learn how to respond to center clients about research-related questions. - Learn how self-compassion could be applied to center programming. - Co-authorship. Publications demonstrate to our members that our organization uses evidence-based information to inform our practice. - Researchers should share pre-prints or e-prints of manuscripts/publications stemming from this research with the center. |
| 2 | What does a “meaningful contribution” mean / look like / feel like to you | - Setting a Terms of Reference for our work together (this discussion). - Researchers should respect that our center is not just a vehicle to access research participants. |
| 3 | How can we support you in this collaboration? | - Researchers should provide reminders of progress at the start of meetings (e.g., who we are, what we are doing, and why). - Researchers should create a poster or infographic about the study and eventual results to post at the center and distribute in the newsletter. - Researchers should share a brief presentation of the study results to staff. - Researchers could organize an educational session on the study for center clients. |
| 4 | What are your preferred methods of providing feedback to the research team? | - Verbal feedback in a meeting - Type of feedback depends on a type of information (e.g., dates, times, signing a document can be done on email) - Feedback on documents – verbal discussion - PowerPoint updates and discussion - When asking community partner to review the grant, the researchers should provide an overview of key points in each section and ask for specific feedback. - Meetings are opportunities to discuss and ask questions. Community partner may need reminder of which sections have been read already. - Researchers should send meeting materials ahead of time for community partner to review before meetings. |
| 5 | How often would you like to meet? | - Meet at each important milestone in grant development – researchers to define specific milestones. - Meetings may become more frequent closer to the grant submission deadline. |
| 6 | How would you like to receive progress updates? | - Researchers should send updates only when there are new developments to share. - A few emailed bullet points with each grant milestone would be helpful. |
| 7 | Is there anything else you would like to add? | - If the researchers choose to recruit our clients into the study, they should first host an information session at our center to provide background information about the study to potential participants. - Community partner should have an opportunity to provide advice on participant-facing materials. - Researchers should provide an overview of opportunities for involvement throughout the research cycle to community partners – if grant is funded. |

| **Supplemental Table 4**  Rural Community Partner Terms of Reference | | |
| --- | --- | --- |
| Item # | Question | Community Partner Response |
| 1 | What are your goals for this partnership? | - Offering well rounded programming in cardiac rehabilitation - Self-compassion as a skillset to offer participants to increase behaviour change and adherence. - Lasting change after the program is over in physical activity and lifestyle. - Manuscript preparation (proof-reading, writing). - Data collection and analysis. - Recruiting research assistants and staff to contribute to the study. - Recommendations for support staff to become involved. - Co-authorship on resulting publications. |
| 2 | What does a “meaningful contribution” mean / look like / feel like to you | - Researchers should share the information needed to create improvements in self-compassion. - Researchers should advise on the minimum amount of time needed to create meaningful change in self-compassion. - Researchers should advise which materials are needed to create improvements in self-compassion. - Researchers should share more about needs of the project and whether participant recruitment would occur at the rural center. |
| 3 | How can we support you in this collaboration? | - Researchers should provide information about the training recommended for a self-compassion facilitator. - Researchers should test a facilitator training package in the first iteration of the project. - Researchers should provide information about sample size, study documents, inclusion criteria, and exclusion criteria. |
| 4 | What are your preferred methods of providing feedback to the research team? | - Either meetings or email feedback – depending on availability. |
| 5 | How often would you like to meet? | - Community partner open to meeting as needed – recognition that in the planning stages of the grant there will be a need for more frequent meetings. - Community partner would like an opportunity to review the grant before submission. |
| 6 | How would you like to receive progress updates? | - Email updates with milestones |
| 7 | Is there anything else you would like to add? | NA |
